# Living well? The unintended consequences of highly popular commercial fitness apps through social listening using Machine‐Assisted Topic Analysis: Evidence from X

**DOI:** 10.1111/bjhp.70026

**Published:** 2025-10-22

**Authors:** Florence Sheen, Lucy Porter, Trisevgeni Papakonstantinou, Maria Ceka, Paulina Bondaronek

**Affiliations:** ^1^ School of Sport, Exercise and Health Sciences, National Centre for Sport and Exercise Medicine Loughborough University Loughborough UK; ^2^ Centre for Behaviour Change University College London London UK; ^3^ Division of Psychology and Language Sciences, Department of Experimental Psychology University College London London UK; ^4^ Psychology Department University of Westminster London UK; ^5^ Institute of Health Informatics University College London London UK

**Keywords:** fitness apps, machine learning, qualitative data, social listening, unintentional consequences

## Abstract

**Objectives:**

Use artificial intelligence–Human collaboration to investigate the unintended consequences of the most popular commercial fitness apps through social listening via X (formerly Twitter) posts.

**Design:**

Machine‐assisted topic analysis (MATA).

**Methods:**

X posts (*n* = 58,881) referring to the five most profitable fitness apps were collected via application programming interface and filtered for negative sentiment, resulting in 13,799. MATA was used to generate a structural topic model. This organized the data into topics and provided 20 representative posts per topic for further qualitative analysis, informed by a thematic analysis approach.

**Results:**

Six topics were generated by machine analysis and subsequently retained as independent themes during human analysis. These reflected key challenges and unintended consequences of using commercial fitness apps, including negative psychological and behavioural impacts. These centred around the challenges of quantifying real‐world activities, implications for accuracy, difficulties associated with achieving algorithm‐set goals, and subsequent negative impacts on emotions, motivations, and engagement with apps and health behaviours more generally.

**Conclusions:**

This study highlights the negative behavioural and psychological consequences of commercial fitness apps as reported by users on social media. Our findings suggest that these may undermine the apps' potential to promote health behaviour change and well‐being. This highlights the need for a more user‐centred app design based on psychological theory, prioritizing well‐being and intrinsic motivation over rigid, quantitative goals.

**Statement of Contribution**

**What is already known on this subject?**

- Fitness apps are widely used for self‐monitoring health and behaviours, promoting accessibility and convenience.
- Concerns about their unintended consequences, such as undermining intrinsic motivation and potential links to disordered eating behaviours, have been noted but are underexplored.
- Social media data provides valuable insights into user experiences, including usability, safety, and emotional impacts.

**What does this study add?**

- Highlights negative psychological and behavioural impacts of fitness apps as reported by users.
- Identifies challenges with app accuracy, unrealistic goals, and overreliance on quantification.
- Demonstrates the potential of AI‐human collaboration to analyse large‐scale social media or qualitative data.

## INTRODUCTION

Digital health apps present substantial opportunities to support people to live well, allowing remote intervention delivery, self‐monitoring or self‐administered interventions (Patel et al., 2021; Philippe et al., 2022), and the potential for greater cost‐effectiveness compared with traditional face‐to‐face formats (Gentili et al., 2022). However, many apps do not appear to use evidence‐ or theory‐based approaches for supporting health behaviour change (Mangone et al., 2016; Portenhauser et al., 2021; Tofighi et al., 2019; Tucker et al., 2021; Zhou et al., 2024). While evidence‐ and theory‐based apps do exist, they may be associated with a higher price point (Cowan et al., 2013) and are no more likely to be used than other apps on the market (Tucker et al., 2021). When it comes to using apps to support health‐promoting behaviour change (such as increasing physical activity or modifying diet), the behaviour change content within these apps tends to rely on a small selection of behaviour change techniques (BCTs), such as prompts or cues, goal setting, feedback, and monitoring (Aguiar et al., 2022; Antezana et al., 2020; Bondaronek et al., 2018; Dunn et al., 2018). A previous review of apps for physical activity and diet change found that the majority centred around behavioural tracking (Bardus et al., 2016). A previous review of apps for physical activity and diet change found that the majority centred around behavioural tracking (Bardus et al., 2016).

Mechanisms of change (or absence of change) can be elucidated through investigating user experiences to understand the effectiveness of apps in changing behaviour. For example, a recent study on one AI‐powered fitness app found that while there were 15 BCTs incorporated into the app (most of which were positively received as helpful and acceptable by participants), there were also outstanding concerns related to low intrinsic motivation for continuing physical activity (Kuru, 2024). This may be associated with the types of BCTs commonly incorporated into these apps; many of the most common BCTs used in fitness apps (such as goal setting, self‐monitoring of behaviour, and feedback on behaviour) primarily target motivational mechanisms of behaviour change such as intentions and goals (Michie et al., 2011). Perhaps unsurprisingly, a systematic review of self‐monitoring intervention literature found that a primary theme of this research focuses on motivation as a key driver of behaviour change (Feng et al., 2021). While motivation is certainly an essential driver of behaviour (Michie et al., 2011), there are a number of potential issues with this relatively narrow approach to changing health behaviours. First, the Capability‐Opportunity‐Motivation model of Behaviour (COM‐B; Michie et al., 2011) stipulates that in addition to motivation, the target audience must have the capability to undertake the behaviour (i.e., relevant knowledge and skills), and they must also have the opportunity to do so (i.e., physical and social environments that are conducive to the behaviour). On this latter point, it is widely recognized that structural issues such as the accessibility and affordability of resources (e.g., nutritious food, gas and electricity for cooking, exercise clothing and equipment), the spaces within which people live (e.g., access to kitchens or safe spaces to exercise), and having the time to engage in health‐promoting behaviours, such as shopping, cooking, and exercising are fundamental contributors to health inequities (Alliott et al., 2022; Katangwe‐Chigamba et al., 2024; O'Donoghue et al., 2018). As a consequence, interventions that require a high level of agency from individuals (such as fitness apps) have been critiqued for being more likely to widen inequalities (Adams et al., 2016).

Second, some evidence suggests that self‐monitoring interventions may sometimes have unintended negative impacts on behavioural motivation. For example, Etkin (2016) found that while measurement (i.e., self‐monitoring) increased how much of an activity people did, it also decreased their enjoyment of that activity. Thus, fitness apps may undermine intrinsic motivation to meet health goals, theorized to be particularly important for health behaviour change (Ryan & Deci, 2000). Self‐determination theory, which has been extensively applied to explain health and fitness behaviours such as physical activity (Ryan et al., 2009), theorizes that intrinsic motivation arises when autonomy, competence, and relatedness needs are met (Deci & Ryan, 2000). In the context of fitness interventions, this may translate into a need to encourage user choice and ownership over fitness goals, support user confidence in their skills and capabilities to achieve fitness goals, and provide opportunities for social connectedness (Molina & Myrick, 2021). While some studies have found positive impacts and opportunities for fitness apps to promote intrinsic motivation (e.g., Fu et al., 2023), no research to date has explored the potential negative impacts that widely used fitness apps may have on such drivers.

As well as considering the impacts of popular fitness apps on desired behaviour change (such as increased physical activity and more balanced nutritional intake), it is also important to consider the potential for negative unintended consequences of fitness apps. Previous research has suggested that a focus on monitoring calorie consumption and expenditure may also encourage cognitions and behaviours that align with disordered eating patterns (e.g., exercising to ‘make up’ for food consumed or becoming ‘obsessed’ with calorie intake) (Levinson et al., 2017; Linardon & Messer, 2019; Simpson & Mazzeo, 2017a; Solbrig et al., 2017). For example, Orji et al. (2018) reported concerns from potential app users that self‐monitoring and calorie tracking would lead to or exacerbate eating disorders, while Eikey (2021) recorded the experiences of fitness app users with eating disorders, who reported that dietary rigidity and a fixation with numbers got worse when using such apps. The association between app use and eating disorder symptomatology has also been identified in quantitative research (Simpson & Mazzeo, 2017b). However, much of this research has been carried out with a relatively narrow group of participants, namely, college students and those with eating disorders; the extent to which this generalizes to other app users who do not actively engage in research studies is less certain. These gaps not only highlight potential safety issues but also represent a missed public health opportunity (Simpson & Mazzeo, 2017a). However, much of this research has been carried out with a relatively narrow group of participants, namely, college students and those with eating disorders; the extent to which this generalizes to other app users who do not actively engage in research studies is less certain. These gaps not only highlight potential safety issues but also represent a missed public health opportunity.

While some studies have explored these issues from the perspective of users (e.g., Kuru, 2024), qualitative studies on the topic have necessarily been relatively small‐scale and only report the perspectives of those who actively engage in research. Compared with the wider population of fitness app users who engage with commercial apps rolled out on the major app stores, very little is currently known about the impacts of these interventions on behaviour change and the mechanisms of behaviour change. In the absence of empirical studies evaluating health apps, social media can provide valuable insights into the experience of thousands of users providing feedback on health apps, with the potential to uncover important usability, safety, and efficacy concerns that developers might not have considered. Lagan et al. (2020) highlighted the need for a public, interactive, and accessible approach to data collection when it comes to app evaluation, for which social media presents a promising avenue. X (formerly Twitter) provides a rich source of real‐time data reflecting users' opinions and attitudes through their interactions. Unlike traditional self‐reports, this data offers an unobtrusive way to observe genuine expressions and reactions, including responses to critical events. The platform's vast daily output on diverse topics presented a valuable opportunity for researchers to analyse extensive public discourse. The application programming interface (API), which provided structured programmatic access to the platform's data including content and meta‐data, allowed researchers to access and utilize data on users' platform interactions (e.g., X posts or ‘likes’; note, as of February 2023, the API is no longer accessible to researchers). By employing a hybrid analysis approach, termed ‘Machine‐Assisted Topic Analysis’ (MATA), which integrates natural language processing (NLP) with human qualitative analysis (Bondaronek et al., 2023; Towler et al., 2023), the current study aimed to gain novel insights into user experiences of fitness apps from vast amounts of social media data. While NLP algorithms enhance efficiency and scalability in processing large datasets, human analysis provides essential context and depth, ensuring a comprehensive understanding of the data. Combining these methods helps achieve a more accurate and meaningful interpretation of user feedback, benefiting digital healthcare solutions. The proof‐of‐concept for this approach was conducted in a previous study within the context of a public health emergency; MATA was used to analyse user feedback from 37,914 English adults on the NHS Test and Trace Service during COVID‐19 (Bondaronek et al., 2023). The study revealed important insights about the functioning of the system and offered recommendations for improvement. Additionally, a recent methodological study demonstrated that the analysis provided by MATA is comparable to human analysis in terms of accuracy but is also significantly more efficient (Towler et al., 2023).

In summary, there is currently minimal exploration of potential negative consequences of using fitness‐tracking apps. This study aims to employ a systematic artificial intelligence–human collaboration method to explore potential negative consequences of the most popular commercial fitness apps through social listening—analysing user experiences expressed in their X posts (colloquially referred to as ‘tweets’). Using MATA, the study seeks to understand potential issues, negative consequences, and side effects of these highly popular and highly grossing publicly available apps.

## METHODS

### Study design

**FIGURE 1:**
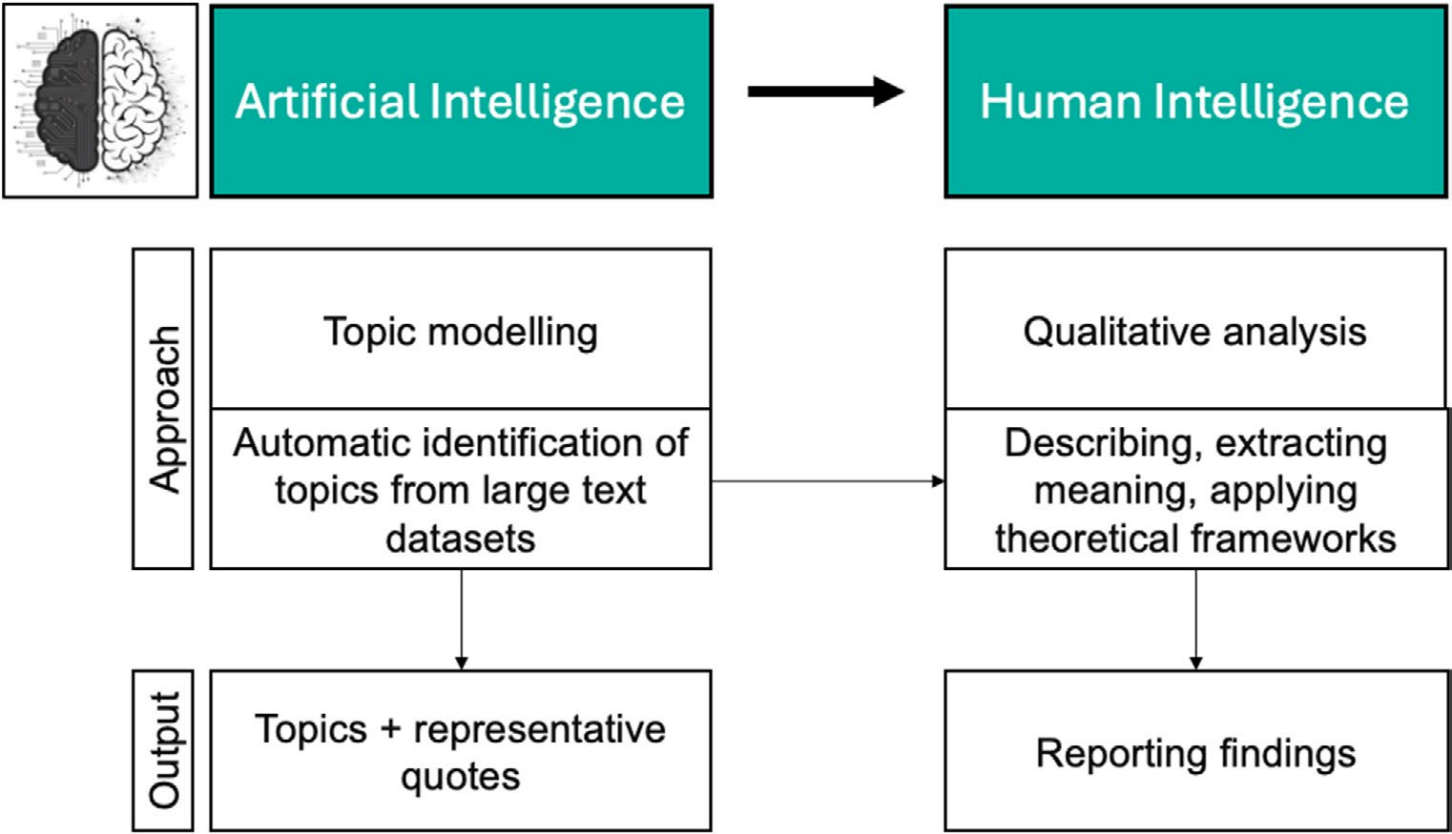
Machine‐assisted topic analysis process.

The current study used a hybrid analysis approach, termed ‘Machine‐Assisted Topic Analysis’ (MATA) (Figure 1), which integrates machine learning with human qualitative analysis (Bondaronek et al., 2023; Towler et al., 2023). It includes topic modelling to analyse large text dataset to identify topics and generate representative quotes. This computational step leverages the speed and accuracy of AI to process extensive datasets, producing outputs that serve as the basis for qualitative analysis. Human intelligence then takes over, with qualitative researchers describing, extracting meaning, and applying theoretical frameworks to topic modelling output, resulting in qualitative synthesis. It includes topic modelling to analyse large text dataset to identify topics and generate representative quotes. This computational step leverages the speed and accuracy of AI to process extensive datasets, producing outputs that serve as the basis for qualitative analysis. Human intelligence then takes over, with qualitative researchers describing, extracting meaning, and applying theoretical frameworks to topic modelling output, resulting in qualitative synthesis.

### Sample

Social media posts from X that referred to commercial fitness apps were eligible for inclusion in this study. Apps were excluded if they did not have stand‐alone functionality (e.g. required the purchase of additional equipment) and if they did not accommodate English‐speaking users. The top 5 highest grossing apps worldwide, as of March 2022 (Statista, 2023), that met these criteria were selected to refine the scope of the study (MyFitnessPal: Calorie Counter; Strava: Run, Ride, Swim; WW (formerly Weight Watchers); Workouts by Muscle Booster; Fitness Coach & Diet: FitCoach). Social media posts from X that referred to any of these five commercial fitness apps were eligible for inclusion in this study.

We scraped X posts mentioning the relevant apps using X's official API with an academic licence, as implemented through the R package ‘academictwitteR’ (version 0.3.1) (Barrie & Ho, 2021). The keywords used included the application's name paired with the words ‘app’, ‘application’, and ‘tracker’ where applicable. We also searched for posts mentioning the handle (or username) of official app accounts. File S1 shows the keywords used; the code and the detailed output can be found in the Open Science Framework (https://osf.io/ruvaf/). The initial search yielded 58,881 X posts. We filtered by negative sentiment using the ‘bing’ lexicon through the ‘tidytext’ package, version 0.3.1 (Silge & Robinson, 2016). Table 1 presents a breakdown of the sample of posts analysed.

**TABLE 1:** Number of sample posts analysed split by source.

Commercial fitness app	*N*
MyFitnessPal: Calorie Counter	8464
Strava: Run, Ride, Swim	2264
WW (formerly Weight Watchers)	2902
Workouts by Muscle Booster	11
Fitness Coach & Diet: FitCoach	158
Total	13,799

### Data analysis

We applied a machine‐assisted topic analysis (MATA) approach, which combines structural topic modelling (STM) with human qualitative analysis. STM was used to generate interpretable clusters of co‐occurring words and representative quotes, which were then thematically analysed by researchers to develop human‐derived themes through iterative coding, review, and refinement The methodology of MATA is also described in Bondaronek et al. (2023) and Towler et al. (2023).

### Machine analysis process

#### Model selection and preparation

To determine the optimal number of topics for our STM analysis, we fitted models with topic numbers ranging from 5 to 40 and evaluated them using several diagnostic criteria: semantic coherence, exclusivity, residuals, and held‐out likelihood. This selection process involved assessing trade‐offs among these metrics in relation to the specific objectives of our analysis.

Semantic coherence, following Mimno et al. (2011), measures how frequently the top words in a topic co‐occur within documents and is considered a key indicator of interpretability. However, models with high semantic coherence alone can overfit to frequent or generic words. We further evaluated held‐out likelihood using a document‐completion method (Asuncion et al., 2025; Wallach et al., 2009) where portions of documents are withheld during model fitting and then used to assess predictive performance. This is similar in spirit to cross‐validation and serves as an estimate of a model's generalizability. Residuals were examined to assess model fit, based on the overdispersion diagnostics outlined in Taddy (2012). Overdispersed residuals indicate that additional topics may help better account for variation in the data. Overdispersed residuals indicate that additional topics may help better account for variation in the data.

Diagnostic plots were generated using the searchK and plotModels functions to visualize how these metrics evolved across different topic counts. We ultimately selected a 10‐topic solution as it offered a strong balance across higher coherence, exclusivity, and held‐out likelihood with lower residuals, while yielding interpretable and distinct topic clusters aligned with our analytical objectives.

Following this, we applied STM, a probabilistic topic modelling technique that uncovers latent thematic structures in text corpora by identifying clusters of co‐occurring words (topics). STM was implemented using R (version 3.5.2) with the quanteda (version 2.0.1) and STM (version 1.3.3) packages. The data preparation involved cleaning the text by removing punctuation, symbols, and numbers, then converting the text into token units. Stop words were removed, and stemming was applied to reduce words to their root forms, which streamlined processing and analysis. In this instance, no structured data was used as covariates in the STM.

#### Model output

The STM output included two primary elements: lists of weighted words and representative quotes. Each topic was characterized by the highest probability words (words with the highest probability within each topic) and FREX words (words that are both frequent and exclusive, distinguishing each topic from others). Additionally, the model generated a list of 20 representative quotes for each topic, providing contextual insight. Examples of the outputs are detailed in the results section.

### Qualitative analysis process

First, all data was anonymized (e.g. ‘X’ account names, or ‘handles’, removed). In MATA, ‘topics’ refer to machine‐generated outputs produced via STM, whereas ‘themes’ refer to the human‐derived categories developed through qualitative interpretation of these topics. Following the generation of STM outputs, a qualitative analysis was conducted, informed by a thematic analysis approach. Thematic analysis typically involves familiarizing yourself with a qualitative dataset, coding meaningful instances, appraising these codes, and organizing them into themes, which are reviewed and reassessed until final themes are produced (Braun & Clarke, 2006, 2019). We outline below the steps we took to reach refined final themes.

#### Labelling representative tweets within each topic and developing descriptive labels for topics

Researchers MC and JB independently analysed the data. Once familiarized with the data associated with each topic generated by the structural topic model (STM), the researchers provided brief descriptions of each representative tweet within the topics to identify patterns related to users' experiences.

#### Collaborative review

Following the completion of this process, MC and JB convened to discuss the issues represented within each topic and agree on the descriptive labels for the topics. In instances of discrepancies between MC and JB, a third researcher, PB, facilitated discussions to resolve differences, ensuring a thorough and consensus‐driven approach to data interpretation. The topic description labels were then submitted to a final round of refinement through discussion between PB, LP, and FS.

#### Theme development

LP and FS expanded upon and refined these initial topic descriptions, taking an approach informed by thematic analysis that involved familiarization with the 20 representative quotes per topic, examining perceived patterns within the labelled topics, and updating or refining the detailed topic descriptions accordingly. LP and FS also looked to group topics together into broader themes where appropriate (discussed with PB). This involved iterative review and refinement of topic descriptions, as well as the selection of illustrative quotes.

## RESULTS

**FIGURE 2:**
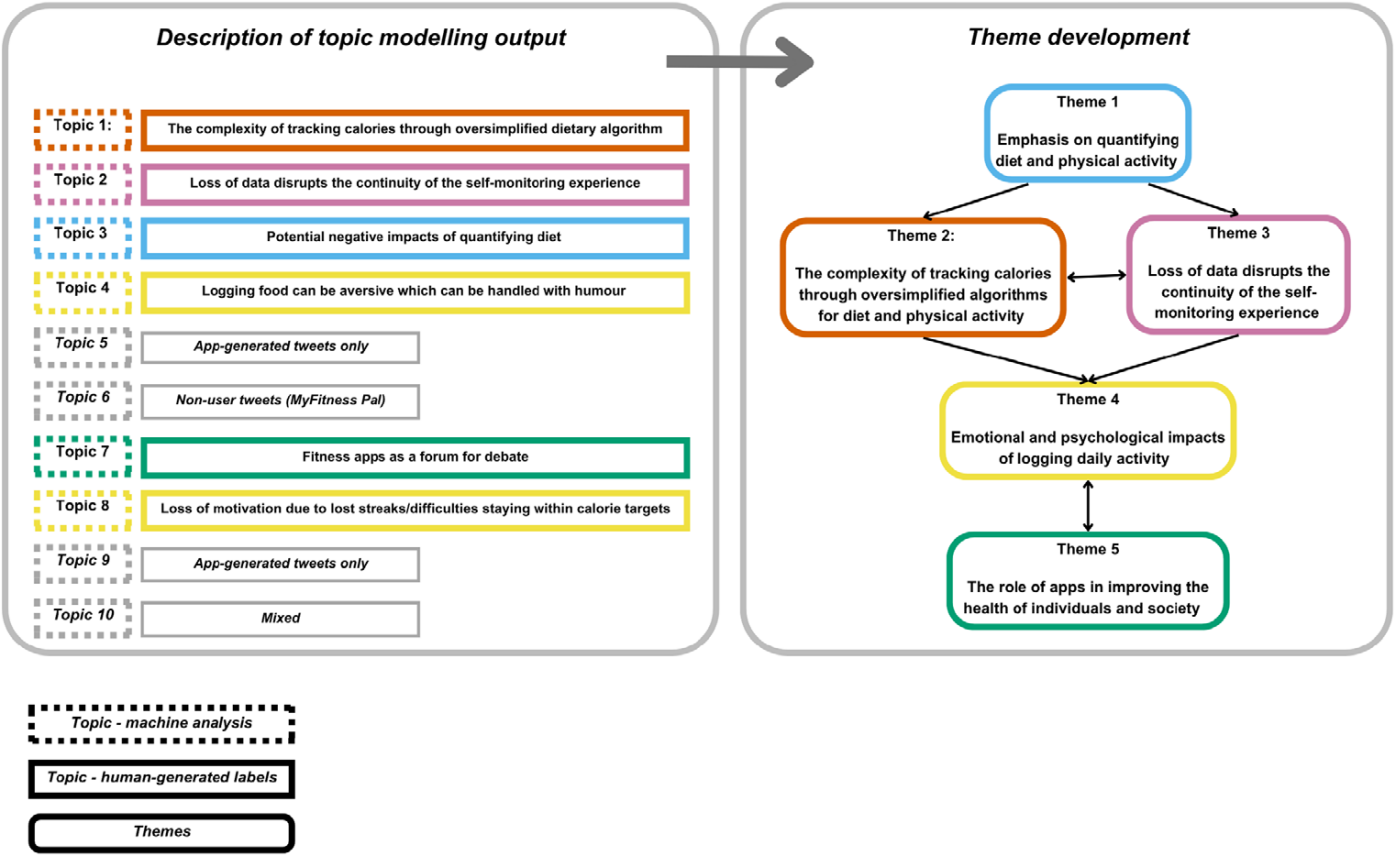
Topic modelling analysis and thematic analysis mapping.

The output of the topic modelling analysis yielded 10 individual topics for human analysts to review (see Figure 2). Of these, six were retained for further analysis and four were excluded: two topics contained automated posts generated by the apps on behalf of users to report weight lost in a standardized format (Topics 5, 9), another contained automated posts from the app support teams (Topic 6), and one topic contained a mix of posts that were already covered by the included topics (Topic 10) (see Appendix S2). Upon interpretation, LP and FS retained four individual themes and combined two machine‐generated themes into one theme, resulting in a total of five themes. These are described below.

### Theme 1: Emphasis on quantifying diet and physical activity

A typical function of commercial fitness apps is to quantify the amount of food users consume daily. Users would use the apps to report the foods they had consumed, which would then be broken down into macronutrients (grams of fat, carbohydrates, protein) or summarized in terms of how many calories the meal(s) were estimated to contain. Sometimes the individual would report a specific calorie intake goal they were trying to achieve, typically a reduction in calories consumed, and food would be referred to in light of these calorie or macronutrient goals.

…If I was hungry between my first meal and dinner I would snack on a piece of fruit + a small handful of almonds, or peanuts, or a cup of full fat yogurt (full fat yogurt makes me feel full). I track the calories for it all in MyFitnessPal and tried to stay under 1600.

For the whole lot: 2420kcal, 56g fat, 298g carbs and 180g protein, according to MyFitnessPal.

The app is my fitness pal. Basic meal breakdown: B: 5 eggs, cereal, berries, toast L: 2 cups diced potatoes, beef, veggies, rice, and a sandwich D: A box of mac and cheese and a can of beans Two 600−calorie protein shakes and random slices of bread between meals. Ugh…

There was some minor evidence of struggles around keeping within calorie goals, including some users reporting eating certain foods to feel fuller to facilitate meeting their calorie consumption goals. Others used humour to illustrate the difficulty in meeting these goals, with the latter quote below also indicating potential underreporting (albeit through the assumed use of humour).

So…how do I explain to My Fitness Pal that I'm rolling taco−size wraps in the frying pan and smearing leftover hoisin on them because I'm craving carbs? #weightlossjourney

#dieting #dietsad

what I say to myfitnesspal ‘I had a small portion of chips & sausage’. what I did irl− two sausages mushy peas deep fat fried chips…

The remaining topics identified in this analysis further explore the challenges and negative consequences of quantifying diet and fitness in this way.

### Theme 2: The complexity of tracking calories through oversimplified algorithms for diet and physical activity

Following on from the emphasis on quantifications, we observe potential issues with the accuracy of quantifying diet and fitness in this way as well as users' concerns and confusions that may arise from this. These apps rely on algorithms that do not reflect the flexibility and messiness of real life or account for individual circumstances and differences, as indicated in users' social media posts. Users report issues with accurately tracking their calories through fitness apps and that the apps did not always account for calorie expenditure in a consistent way or in alignment with users' expectations (e.g., for non‐exercise activities such as breastfeeding).

Can any breastfeeding mama's tell me how to add breastfeeding in my fitness pal? I looked up how but it didn't subtract my calories.

Users also directly referred to concerns about accuracy, which may have arisen from posts they had viewed elsewhere on social media:

help i just saw a reel of a woman saying that adding on MyFitnessPal the exercise calories are unnecessary bc the apo puts weird inexact amounts WICH MEANS I'VE BREN COUNTING ANYTHING BAD I HAVE NO CLUE HOW MUCH I BURN HELP

Users also encountered difficulties with unclear advice from the app when entering their food intake:

I use Alpro yogurt, but when I enter the product on my fitness Pal app, it says my goal should be zero polyunsaturated and mono unsaturated, confused

In addition, there was surprise from some users around the recommendations made by these apps. In particular, a few users highlighted or advised using these apps only to track calories consumed, not calories burned. Otherwise this could lead to a recommended calorie deficit that was both unachievable and unhealthy, with users referring to experiences of ‘starving too often’ while trying to maintain the recommended calorie deficits.

So my fitness pal says if I want to reach my goal weight I need to consume −700 (negative 700) calories a day

My Fitness Pal should be used for tracking calories ONLY. If you allow it to prescribe your calories you'll end up with a deficit that's unachievable, unsustainable and very unhealthy. You could also starve to death ….

### Theme 3: Loss of data disrupts the continuity of the self‐monitoring experience

In a similar vein, users also described issues related to the accuracy of tracking as a result of technical challenges and malfunctions, further demonstrating the difficulties of quantifying real‐life activities using fitness apps. These included difficulties syncing data across devices and apps:

Help When I sync TR with Garmin Connect and Strava and Garmin Connect also uploads to Strava (outdoor rides) should I worry about duplicates in TR?

Users also described instances of data loss resulting from technological malfunctions and issues such as cyber‐attacks, software crashes, and battery loss. Some data loss also occurred due to human error.

Argh, got a PB for a half−mara in training and my phone died at the end, so none of it recorded to @strava. Shouldn't matter… (but does).

No its gone I looked for it. Remember Garmin is connected to Strava so I deleted the run …………. after I took the picture of the run. I was deleting pictures but ended up deleting the run. The pain eish

As a result, some users questioned the accuracy of their tracking results, especially when results for the same activity (e.g., cycle ride, swim, or run session) varied according to different apps and devices:

Probably doing something wrong but exported the .gpx file for 2022 ride london from ride with GPS 102.6 miles 3600ft of climbing to Strava and it's 89 miles and 1200 feet of climbing, why?

Users took to social media to discuss these problems with other app/device users. In the case of challenges with syncing data across devices, other users suggested solutions such as waiting for a period of time before trying again, connecting their devices to a personal computer, and manually downloading and uploading files between devices. There was also some minor evidence that these challenges may contribute to negative emotions such as stress and anxiety, with occasional references to feelings of ‘desperation’ and ‘worry’.

Out of desperation I linked my Sigma to Mapmyfitness, I record my ride then export it as a tcx file to Strava.

### Theme 4: Emotional and psychological impacts of logging daily activity

This topic moves away from challenges of data accuracy and focuses instead on the emotional and psychological consequences of quantifying fitness. This topic can be conceptualized as having two subthemes: logging dietary intake can be aversive (shame, irritation, guilt), which can be handled with humour and loss of motivation due to lost streaks and difficulties achieving targets.

The first subtheme mainly contained posts that referred to reminder notifications sent by My Fitness Pal, prompting users to record their daily food intake. Specifically, users expressed being ‘sick of’ or ‘pissed off with’ the app, indicating that the notifications became aversive over time. This appeared to be related to the users' perceptions of the quality of their diet and not wanting to record meals that were unlikely to meet the nutrition targets set by the app, for example:

Hate when my fitness pal asks why I haven…t logged my dinner ! Haven…t logged my dinner mate cause I…ve ate a spice bag and milled a tub of Ben and Jerry…s leave me alone!.

Many of the posts in this topic took a similar format, whereby the user shared an interaction between the app notification and the user's thought processes in the form of a script:

My Fitness pal: Hey log your breakfast! Me: cup of coffee, cup of coffee, cup of coffee My Fitness pal: haha okay, but maybe some food? Me: CUP OF COFFEE, CUP OF COFFEE, CUP OF COFF… My Fitness pal: k, sorry we asked

myfitnesspal: …you haven…t logged your breakfast for today ……. yea because breakfast was three pieces of stale bread, half a cigarette i found on the dorm steps, and a bottle of cranberry juice

Many of the posts in this topic were interpreted as taking a comedic tone, meaning that they may not depict users' real‐life food and drink (and tobacco/drug) intakes with full accuracy. Nevertheless, there were similar themes of aversive emotional responses to the app's notifications asking users to log their food intakes. Some of these indicated potential shame responses, for example:

I just got a notification from my fitness pal reminding me to log my dinner for today but I don't want to bc I'm ashamed I just ate dominos.

In a slightly different vein, one user also indicated frustration that the app had sent a notification reminding them to lower their sugar intake after they logged eating a banana, demonstrating that these negative human‐app interactions are not restricted to logging foods that are traditionally seen as ‘unhealthy’ or categorized as ‘HFSS’ foods:

Pissed of with My Fitness Pal awready. Gieing me grief for adding a banana as part of a lunch. ‘StAy BeLoW 56G oF SuGaR’ a fucking banana. Fuck off.

The second subtheme mostly contained posts that centred around the emotional and behavioural consequences of missing targets, particularly with regards to lost motivation. This goes beyond the initial frustration or shame described by users in the previous subtheme by demonstrating the negative consequences that these experiences have on users' motivation. Some described how disappointment at slow progress or horrified surprise at the nutritional content of favourite foods could lead to counterproductive behavioural responses, such as giving up and returning to previous dietary habits or avoiding logging certain foods in the apps:

How disappointing is it when you smash gym and MyFitnessPal for a day and there…s no difference …. back to eating lotus biscoff spread out of jar.

Unless you want ruin a large part of your life…. Do NOT put Percy Pig sweets into MyFitnessPal. WHAT.THE.ACTUAL.FUCK. No wonder I made it up to 23 stone at one point.I could eat a carrier bag of them. Don…t do it people. I am MORTIFIED. Its PringleGate all over again.

Others indicated that these instances increased motivation for trying harder, however this was framed in terms of negative affect:

I just plugged in my lunch eats into My Fitness Pal…aaaaannnddd I'll be going to the gym later. Also, my hangry ass ate too much. I feel miserably stuffed.

A few posts also described the disappointment of losing ‘streaks’ whereby the user had achieved a particular target for a certain amount of time but missed the target before an important milestone (e.g., 100 days in a row). In these cases, the progress and achievements made in terms of meeting dietary/physical activity targets on a daily basis are not mentioned, with the focus instead being on the loss of the overall streak target.

I was three days away from hitting my 100 day log streak on My Fitness Pal. I missed one day and it's back at zero …

### Theme 5: The role of apps in improving the health of individuals and society

Beyond individuals' relationships with fitness tracking, fitness apps were also discussed in terms of their suitability as a health intervention in wider society. Narratives were often largely supportive of fitness apps and emphasized neoliberal ideas around taking personal responsibility for one's own health and that of their family.

Fox news was just criticizing a weight watchers app for kids. Disagreeing with the apps recommendations. If your kid is 8 yrs old and fat, then its the parents fault!! Any parent that allows their kid to become overweight is an unfit parent and that should be the focus!

One user also indicated support for fitness apps as a public health intervention to reduce body weight among the population, among other approaches such as taxing people according to their body weight:

…Absolutely, then start taxing for each % of bodyfat over 10%…that causes more fatalities than covid ever will. Have the govt buy MyFitnessPal and make monthly weigh−ins mandatory. A new tax along with the requisite bureaucratic hiring binge does not solve every problem!!!!

Interestingly, discussions of personal responsibility also extended to the potential negative impacts of these apps. For example, one user posited that fitness apps cannot be blamed for contributing to eating disorders as ‘so many people use that app in a healthy way’:

some edtt girlys were blaming MyFitnessPal for there eds like be for real So many people use that app in a healthy way, no way in hell is it their fault Same with all the cringey edtt girls blaming chloe ting and daisy keech for there eds stop omfg. Cringey!!! Annoys me sm ugh

## DISCUSSION

This study is the first to investigate potential negative consequences of popular commercial fitness apps using MATA as a social listening tool to analyse social media posts. The combination of human and machine learning analysis resulted in the generation of five themes focusing on users' experiences of the five highest grossing commercial fitness apps worldwide. These themes highlighted how the self‐monitoring function of many fitness apps leads to an emphasis on quantifying diet and physical activity and the setting of (at times, unrealistic) goals that are expressed as numeric targets (e.g., intake of macronutrients, or calories consumed versus expended; Theme 1). However, the accuracy of such quantitative tracking can be compromised by oversimplified algorithms that do not fully account for users' individual needs and activities (Theme 2) and by technological malfunctions and human errors when using the apps and associated hardware (Theme 3). Unsurprisingly, users may experience a range of negative emotions in response to the challenges of working towards potentially unrealistic targets, reporting feelings of shame at their supposed transgressions and irritation at the notifications sent by these apps. These experiences, as well as the experience of losing a ‘streak’ for consistent performance, appeared to impact users' motivation and behaviours; some appeared to give up when targets could not be obtained, whereas others seemed to maintain perseverance driven by negative emotions (Theme 4). Finally, Theme 5 reported wider discussions on the suitability of these apps as health interventions within society, with a particular focus on narratives of personal responsibility.

### Quantification of real‐world activities and implications for accuracy

One of the key issues arising from this analysis centres around commercial fitness apps placing primary focus on quantifying diet and physical activity. Interestingly, one of the topics excluded from this analysis for being out of scope perfectly illustrates this point: it was a topic consisting entirely of automatic, app‐generated posts that followed the format of ‘[User] lost X lbs since last weighing in!’. However, such a quantitative‐led approach cannot fully capture the unique experiences and complexity of daily life, which can subsequently lead to inaccuracies in progress reporting. While previous research on the reliability of self‐report measures of diet and physical activity has focused on the inaccuracies that can arise from human error in data entry (e.g., as a result of social desirability bias or forgetfulness (Subar et al., 2015), or simply due to the complexity and resource‐intensive nature of monitoring accurate caloric intake), the current findings highlight that further inaccuracies may be generated by commercial apps themselves. While some of these inaccuracies were due to technical malfunction and data loss, the findings of this study indicate that even when fitness apps are fully functional, they cannot offer a fully accurate self‐monitoring experience due to the limited options available for personalization and tailoring. This was illustrated in the case of the app user who could not add their breastfeeding activity into the app to contribute towards calorie expenditure estimates (it is commonly advised that breastfeeding can lead to an additional 500 kcal expenditure per day, including by national sources of health information across the UK, such as NIDirect (2024) and some NHS sources (Buckinghamshire Healthcare NHS Trust, 2024; NHS Lanarkshire, 2024)).

Concerns about inaccuracies in data tracking were also expressed by other users, who took to X to share information about inaccurate calorie reporting by these fitness apps. These concerns are not unfounded, as recent research has confirmed errors in the calorie reporting of apps such as MyFitnessPal (Ho et al., 2024). These findings point to various sources of inaccuracy in self‐monitoring apps, including technological malfunction and data loss, lack of appropriate sufficient tracking options (e.g., as in the breastfeeding case), and incorrect calorie information in app databases. This contributes to a wider evidence base questioning the accuracy of commercial fitness apps for monitoring of dietary intake (Lin et al., 2022). Previous research has also questioned the reliability and validity of various commercial fitness apps as tools to record and assess health‐related outcomes such as physical activity, cardiorespiratory fitness and heart rate (Bouts et al., 2018; Muntaner‐Mas et al., 2019; Silva et al., 2020), although those that collect data via wearable devices have typically been found to provide more reliable and accurate measures (Piccinini et al., 2020; Xie et al., 2018).

### Implications of quantification‐of‐the‐self on human experience and behaviour

The importance of accuracy in self‐monitoring and quantification is underpinned by a second key finding of this study, which focuses on the negative effects of self‐monitoring and quantification on human experiences of health and health behaviour. Users' accounts suggested that the algorithms in these apps set unrealistic goals (e.g., in reference to how many calories should be consumed per day), with some suggesting that adhering to these goals could lead to starvation. The goals set by fitness apps are not based on public health recommendations (e.g. NHS recommendation for daily calorie intake). Instead, these are dictated by the individual user's weight goals, which could lead to unrealistic or unsafe recommendations being given by the app. This, alongside expressions of shock and horror at the calorific value of certain foods (such as ‘Percy Pigs’, a popular sweet/candy in the UK), demonstrates that changing dietary behaviour in line with the goals and recommendations set by fitness apps can be exceedingly difficult.

However, despite these acknowledged difficulties, we also saw evidence of users feeling shame when reflecting on their progress; for example, in a series of comedic and serious posts expressing a desire to avoid logging their food for the day or to input data that was not accurate. This aligns with previous work which demonstrated feelings of stress or self‐disappointment upon not meeting goals (Constantiou et al., 2023). Individuals in the current study also expressed feeling pestered by app notifications, particularly when they had not been able to eat in line with calorie targets, had ‘over’ eaten, or eaten ‘unhealthy’ foods. Perhaps unsurprisingly then, the difficulty and aversiveness of sticking to rigid calorie limits and goals was observed to contribute to a loss of motivation among some users. For example, where individuals faced difficulties in keeping within the targets set by the app (e.g., losing a ‘streak’ or not meeting their daily goal) this appeared to contribute to avoidant behaviours (‘do NOT put Percy pigs into MyFitnessPal’) or complete disengagement (‘back to eating lotus biscoff spread out of jar’). These costs of self‐monitoring dovetail with the findings of Orji et al. (2018), who reported that self‐monitoring had the capacity to demotivate individuals, potentially leading to negative feelings and unhealthy behaviours (e.g., excessive restriction). The implications of these findings, then, is that fitness apps may have the counterproductive effect of ultimately reducing users' motivation and the likelihood of desired health behaviour change, rather than supporting it. The implications of these findings, then, is that fitness apps may have the counterproductive effect of ultimately reducing users' motivation and the likelihood of desired health behaviour change, rather than supporting it.

Where users were motivated to keep striving towards their fitness goals, we also saw evidence that this was driven by extrinsic motivation rather than intrinsic motivation, the latter of which is theorized to be particularly important for health behaviour change (Ryan & Deci, 2000). For example, one user expressed an intention to go to the gym in response to not meeting their calorie intake goals, describing themselves as ‘miserably’ stuffed, indicating that their decision to exercise was driven by negative emotions and the external prompt of the app's feedback. Similarly, those who experienced loss of exercise data due to human error or technological malfunction expressed disappointment and loss of enjoyment in the activity, such as one user who reported losing a record of their personal best time for a half marathon and focused on the disappointment and frustration of this data loss rather than the achievement itself. A similar effect was observed among users who reported losing ‘streaks’ in the app – rather than focusing on the progress that had been made until that point, the user was preoccupied by what had not been achieved. Together, this demonstrates how fitness apps and tracking may undermine intrinsic motivation to meet health goals, which was also demonstrated by Etkin (2016) who found that while measurement increased how much of an activity people do, it also decreased their enjoyment of that activity. This is concerning, given that self‐monitoring as a BCT is primarily employed to increase motivation as a mechanism of behaviour change (Feng et al., 2021). While no theory was explicitly used in the analysis reported here, the findings can be interpreted in light of self‐determination theory (Deci & Ryan, 2000; Ryan et al., 2009; Ryan & Deci, 2000) and the needs for autonomy, competence and connectedness that are theorized to underpin intrinsic motivation. First with regards to autonomy, while users could set their goal weight, the daily calorie intake goals were then automated by the fitness apps themselves, with users finding these to be unrealistic and unobtainable (or risk ‘starv[ing] to death’). Users also sometimes lacked control in terms of logging the activities they deemed important to their health and fitness (such as breast feeding, discussed elsewhere). Second, with regards to competence, the difficulty in achieving such goals (including shock at discovering the calories included in favourite foods and disappointment at not observing desired weight loss despite behaviour change efforts) also appeared to decrease users' motivation in some cases (disappointment at not observing desired weight loss despite behaviour change efforts) also appeared to decrease users' motivation in some cases.

The final theme in our analysis moved away from descriptions of user experiences and instead included X posts from users discussing the role of these apps as a tool for weight management. Users tended to refute criticisms of fitness apps (e.g., that they can exacerbate eating disorder symptoms, as has been demonstrated elsewhere [Levinson et al., 2017; Linardon & Messer, 2019; Simpson & Mazzeo, 2017a]), while ideas of taking personal responsibility for the sensible use of fitness apps and one's own weight (and that of their children) were prominent here. This reinforces neoliberal narratives around weight, which place the responsibility for weight on the individual and overlook the impact of structural and environmental factors such as socioeconomic status and access to nutritious food, contributing to the use of approaches such as blame and fat shaming in obesity policy interventions (Dolezal & Spratt, 2023). These perceptions of blame being situated within the individual align with the findings of earlier themes, particularly in users' expressions of guilt and shame after a lack of success in meeting the strict calorie targets set by the apps, suggesting that such narratives may have become internalized. The internalization of neoliberal narratives and associated fat shame or weight stigma have previously been linked to demotivation and negative impacts on eating behaviour (Rose Spratt, 2021; Tomiyama et al., 2018; Vartanian & Porter, 2016), suggesting a further potential mechanism for the demotivation and disengagement in fitness behaviours reported by users (Theme 5). Additionally, Sax (2021) recently highlighted that the creators of commercial fitness apps are primarily concerned not with maximizing users' health, but with maximizing the conversion of users into profitable users. If users are to shoulder the sole responsibility for their health, it is questionable whether commercial, profit‐driven organizations are the best guardians for their endeavours. Sax (2021) recently highlighted that the creators of commercial fitness apps are primarily concerned not with maximizing users' health, but with maximizing the conversion of users into profitable users. If users are to shoulder the sole responsibility for their health, it is questionable whether commercial, profit‐driven organizations are the best guardians for their endeavours.

## STRENGTHS AND LIMITATIONS

A strength of the current approach is the use of MATA, which allows researchers to investigate the voices of those who may not typically engage with or access research by making traditionally ‘messy’ datasets accessible and usable. This approach allowed us to maximize the strength of artificial intelligence (AI) in efficiency alongside human input to provide valuable insights into the real‐world impacts of these apps. Nevertheless, there are a number of limitations.

First, this was an exploratory study, and while we used sentiment analysis to filter for negative posts, we recognize that sentiment analysis tools have known limitations. For example, seemingly ‘positive’ experiences may still be captured due to the presence of words like ‘loss’, which the algorithm may interpret as negative.

Second, topic modelling identifies clusters of words that frequently co‐occur across documents, based on statistical patterns rather than semantic meaning. As a result, some topics may be less easily interpretable or internally consistent. This underscores the need for careful human oversight during interpretation, as applied in our study through MATA.

Third, future research may benefit from incorporating human input at additional stages of the MATA process—for example, during initial topic evaluation, the merging or splitting of topics, and the assessment of topic coherence—to enhance interpretability and rigour.

Finally, the process of interpreting AI‐generated topics requires structured guidance. Developing shared principles or best practices for conducting MATA would support greater transparency, help identify common challenges, and strengthen methodological consistency in future applications.

## IMPLICATIONS FOR RESEARCH, PRACTICE, POLICY

The use of MATA has implications for research, as it allows the analysis of large‐scale textual data that would otherwise be unmanageable for qualitative researchers. Researchers should consider this method on large qualitative datasets on social media (‘Big Qual’) (Brower et al., 2019; Chandrasekar et al., 2024) to explore positive, negative, and unintended consequences of ‘wellness’ interventions. Given the limited research on the long‐term impacts of apps, additional research should also explore other commercial digital interventions, as well as the long‐term impacts of using commercial digital health on motivation and behaviour change among users, both in terms of intended behavioural outcomes (e.g., reduced calorie/fat/sugar intake, increased physical activity) and in terms of unintended negative consequences (e.g., increased calorie/fat/sugar intake, reduced physical activity, reduced well‐being, reduced motivation, increased eating disorder symptomatology).

For future intervention development, app developers should consider adopting a holistic approach by shifting the focus from merely calorie counting or exercise quantification to increased well‐being as the outcome. Additionally, it is important to explicitly consider potential negative consequences of app features to mitigate the potential harms of ‘wellness’ apps. For example, the dark logic model (Bonell et al., 2015) which provides a structured framework for identifying and addressing potential harms in public health interventions, could be applied to apps to consider and mitigate unintended negative consequences.

Finally, there are important implications for policy. The findings from the social listening exercise based on a large free‐text dataset indicate a prominent focus on calorie counting, which may contribute to detrimental health outcomes on a large scale (e.g., studies showing negative impacts of calorie counting) (Eikey, 2021; Linardon & Messer, 2019; Orji et al., 2018; Simpson & Mazzeo, 2017b; Solbrig et al., 2017). This highlights the need for more stringent regulation of ‘wellness’ apps to ensure their safety and effectiveness. While the regulation of healthcare apps is clearer (e.g., NICE Evidence Standards in the UK [National Institute for Health and Care Excellence, 2021]), there is significantly less clarity and greater ambiguity surrounding so‐called ‘wellness’ apps, with many publicly available apps not subject to formal evaluation. This study suggests that these apps may have unaddressed detrimental effects, emphasizing the necessity for closer scrutiny and regulation.

## CONCLUSION

This study highlights the complex experiences of commercial fitness app users, and the negative consequences they can experience. While these apps are widely used for tracking physical activity and diet, this study reveals that these self‐monitoring features also introduce significant challenges related to algorithmic accuracy, goals that are difficult to achieve, and overreliance on the quantification of progress. As a result, users highlighted numerous negative behavioural and psychological consequences of these apps, including feelings of shame, disappointment, and demotivation, and subsequent disengagement with apps and health behaviours. These findings indicate that features of commercial fitness apps can have negative impacts on well‐being and motivation, with the potential to disrupt health behaviour change for some individuals. The current study highlights the need for better evaluation of commercial fitness apps, as well as more user‐centred and psychologically informed app design that prioritizes well‐being and intrinsic motivation over rigid, quantitative goals – and, indeed, profit.

## AUTHOR CONTRIBUTIONS

**Florence Sheen:** Conceptualization; writing – original draft; writing – review and editing; visualization; validation; formal analysis; project administration; data curation; supervision; software; resources. **Lucy Porter:** Conceptualization; writing – original draft; validation; visualization; writing – review and editing; formal analysis; project administration; supervision; data curation; software; resources. **Trisevgeni Papakonstantinou:** Methodology; investigation; validation; data curation; formal analysis; software; writing – original draft; writing – review and editing; resources. **Maria Ceka:** Writing – original draft; writing – review and editing; formal analysis; validation; software; data curation; resources. **Paulina Bondaronek:** Project administration; conceptualization; investigation; funding acquisition; writing – original draft; methodology; validation; visualization; writing – review and editing; software; formal analysis; data curation; supervision; resources.

## Supporting information


Appendix S1

Appendix S2

## Data Availability

The code and detailed outputs representing the topic modelling and representative posts supporting this study are openly available in the Open Science Framework (OSF) at https://osf.io/ruvaf/. The full dataset for this study is available upon request from the corresponding author.
