# Supplementary material for: Living well? The unintended consequences of highly popular commercial fitness apps through social listening using Machine‐Assisted Topic Analysis: Evidence from X
Source: Br J Health Psychol. 2025 Oct 22;30(4):e70026. doi: 10.1111/bjhp.70026 (PMC12541294; doi:10.1111/bjhp.70026)
Supplement: Supplementary file 1 — Appendix S1 [file BJHP-30-0-s001.docx]

**Supplementary Material 1: Keywords used to gather tweets relating to the 5 top grossing fitness apps**

1. **Coding and Keywords**:

The search terms and keywords used to gather tweets for each of the top five fitness apps:

• **MyFitnessPal**: "myfitnesspal app OR my fitness pal app OR myfitnesspal application OR my fitness pal application OR myfitnesspal"

• **WeightWatchers**: "weightwatchers app OR weightwatchers application OR weight watchers application OR weight watchers app"

• **Strava**: "strava app OR strava application OR strava"

• **FitCoach**: "fitcoach app OR fitcoach application OR fit coach app OR fit coach application OR fitcoach"

• **MuscleBooster**: "musclebooster app OR musclebooster application OR muscle booster app OR muscle booster application OR musclebooster"

2. **Timeframes for Data Collection**:

The start and end dates for the tweet searches:

• **MyFitnessPal**: 2006-03-21 to 2023-01-01

• **WeightWatchers**: 2009-01-01 to 2023-01-01

• **Strava**: 2009-01-01 to 2023-01-01

• **FitCoach**: 2018-10-26 to 2023-01-01

• **MuscleBooster**: 2021-01-01 to 2023-01-01

3. **Languages**:

• Tweets were restricted to English (lang = "en")
