# Supplementary material for: Living well? The unintended consequences of highly popular commercial fitness apps through social listening using Machine‐Assisted Topic Analysis: Evidence from X
Source: Br J Health Psychol. 2025 Oct 22;30(4):e70026. doi: 10.1111/bjhp.70026 (PMC12541294; doi:10.1111/bjhp.70026)
Supplement: Supplementary file 2 — Appendix S2 [file BJHP-30-0-s002.docx]

**Supplementary Material 2: Excluded Topics**

**Topic 5: App-Generated Tweets Only**
**Rationale:** Excluded as the content primarily comprises app-generated updates highlighting users' weight loss progress, which reflect positive app responses rather than user experiences of unintended consequences. Examples:

| *Thanks to @ myfitnesspal and @fitbit I have lost 23.2 lbs since Jan 1st and 32 lbs in total since October 1st 2014. #hardworkpaysoff*  *Weigh−In Wk 6 − Lost 2lb. Total lost − 1 Stone 2lb. Total lost since giving birth − 2 Stone 8lb. I feel amazing thanks to @ww_uk #teamww xx* |
| --- |

**Topic 6: Non-User Tweets (MyFitnessPal)**
**Rationale:** Excluded as the content pertains to customer support responses addressing technical issues, such as glitches or disconnections. These tweets do not reflect unintended consequences experienced by fitness app users. Examples:

*0 Hi ! I will be assisting you here on.I sincerely apologize for the inconvenience caused to you. If you have any concerns with the MPL app, I request you to highlight your specific issue along with the game details so that we can take the (1/2)"*

*Hello this is R from MPL team. MPL strives to ensure fair gameplays for all the players, if you have any concerns with MPL app, I request you to highlight your specific issue so that we can take the necessary steps to resolve them."*

**Topic 9: App-Generated Tweets Only**
**Rationale:** Excluded as the tweets describe app-generated updates tracking users' physical activities and calorie burn. These focus on achievements rather than unintended user consequences.

| *burned 2,898 calories doing 300 minutes of cardio exercises, including "Bicycling, &amp;lt;10 mph, leisure (cycling, biking, b... #myfitnesspal*  *burned 1,326 calories doing 166 minutes of cardio exercises, including "Bicycling, &amp;lt;16 kph, leisure (cycling, biking, b... #myfitnesspal* |
| --- |

**Topic 10: Mixed**
**Rationale:** Excluded as the content is repetitive and overlaps with user concerns in other included topics. The tweets lack uniqueness and do not provide additional insights into unintended consequences beyond issues like tracking difficulties, food shaming, and loss of motivation.

*think i might ignore what my fitness pal is telling me I have left with calories tonight − feeling hungry*

*There's someone I know who did the Afton 25K course in 30 minutes, according to Strava. The record for the Stillwater steps is 13 seconds. Nonsense*

*"3 things that should be left in 2020: − Cellulite being ...ugly..., it...s just a normal physical thing that both genders get. − The scales defining who you are. It...s your gravitational pull to the earth. − My Fitness pal setting everyone...s calories at 1200."*
